# Endothelial Dysfunction in Psoriasis: An Updated Review

**DOI:** 10.3389/fmed.2022.864185

**Published:** 2022-06-10

**Authors:** Panagiota Anyfanti, Anastasia Margouta, Kyriakos Goulas, Maria Gavriilaki, Elizabeth Lazaridou, Aikaterini Patsatsi, Eugenia Gkaliagkousi

**Affiliations:** ^1^Department of Internal Medicine, Papageorgiou Hospital, Aristotle University of Thessaloniki, Thessaloniki, Greece; ^2^Postgraduate Course, School of Medicine, Aristotle University of Thessaloniki, Thessaloniki, Greece; ^3^Department of Dermatology and Venereology, School of Medicine, Papageorgiou Hospital, Aristotle University of Thessaloniki, Thessaloniki, Greece

**Keywords:** endothelial dysfunction, psoriasis, cardiovascular risk, atherosclerosis, circulating biomarkers, vascular biomarkers

## Abstract

Although psoriasis is predominantly a chronic inflammatory skin disorder, epidemiological data provide a solid link between psoriasis, especially in its more severe forms, and increased risk for cardiovascular morbidity and mortality. Apart from the increased prevalence of traditional cardiovascular risk factors, chronic inflammation appears to act synergistically with the underlying process of endothelial dysfunction toward the development of accelerated atherosclerosis, subclinical vascular injury and subsequently, clinically evident cardiovascular manifestations. Endothelial dysfunction is regarded as an early precursor of atherosclerosis with a predictive value for the development of future cardiovascular events. A thorough understanding of the mechanisms of endothelial dysfunction in psoriasis might pave the path for the development of more accurate cardiovascular risk prediction tools and possible therapeutic targets aiming to alleviate the increased cardiovascular burden associated with the disease. The present review summarizes the available evidence about the role of chronic inflammation and other important pathophysiological mechanisms involved in the development of endothelial dysfunction in psoriasis. An overview of studies implementing the most widely applied circulating and vascular biomarkers of endothelial dysfunction in psoriasis patients will be provided, and the impact of systemic psoriasis treatments on endothelial dysfunction and patients’ cardiovascular risk will be discussed.

## Introduction

Psoriasis is a chronic inflammatory skin disease with a reported prevalence of approximately 2% in Europe and North America, triggered and preserved by dysregulated interactions between the innate and adaptive components of the immune system (1, 2). However, cutaneous manifestations are not the only clinical demonstration of the disease. Accelerated rates of cardiovascular complications and major adverse cardiovascular events have been consistently observed in patients with psoriasis (3). Although these patients are often characterized by an unfavorable cardiometabolic profile, increased cardiovascular risk in psoriasis appears to be directly associated with the disease *per se* (1, 2). Pathophysiologically, central to the inflammatory hypothesis of atherosclerosis is the mutual interplay between inflammation and endothelial dysfunction, as has been described in other chronic inflammatory autoimmune disorders (4). Their synergism triggers and augments the sequence of accelerated atherosclerosis, subclinical target organ damage and eventually, clinically evident cardiovascular manifestations (4, 5).

Keeping in mind that endothelial dysfunction is considered as the earliest precursor of cardiovascular disease (CVD) (6), a thorough understanding of this process in psoriasis might commence interventions for the early identification and more effective monitoring of high-risk individuals. Therefore, the present mini review aims to provide an overall assessment of endothelial dysfunction in psoriasis, with emphasis placed on mechanisms that promote endothelial dysfunction and subsequently lead to the increased cardiovascular risk associated with the disease. Studies providing evidence of endothelial dysfunction using established as well as novel markers of endothelial dysfunction in psoriasis will be discussed. Lastly, data regarding effects of psoriasis-specific systemic treatments on endothelial dysfunction will be provided. To this end, a PubMed search was performed to identify relevant articles published in English, using the following medical terms: “psoriasis,” “endothelial dysfunction,” “cardiovascular,” and “atherosclerosis.”

## Cardiovascular Morbidity and Comorbidity in Psoriasis

Psoriasis is associated with multiple cardiometabolic diseases, which are divided into cardiovascular comorbidities and major adverse cardiovascular events (coronary heart disease, ischemic heart disease, myocardial infarction, congestive heart failure, and stroke) all of which contribute to increased cardiovascular morbidity and mortality (7, 8). The strength of this association appears to vary according to psoriasis disease severity (9). Table 1 summarizes relevant systematic reviews and meta-analyses, all of them published within the past decade, supporting the association of psoriasis with conventional cardiovascular risk factors and adverse cardiovascular events.

**TABLE 1:** A synopsis of systematic reviews and meta-analyses investigating the association of psoriasis with conventional cardiovascular risk factors and adverse cardiovascular events.

References	Type of study	Cardiovascular comorbidities	Study population	Key findings
Kaiser et al. (12)	Systematic review and meta-analysis	CAD	14 eligible studies: 1,427 patients with psoriasis and 9,670 controls	Patients with psoriasis (RR = 1.14, 95% CI: 1.04–1.26; *p* = 0.004). For more severe CAD (CCS > 100) the risk was further increased (RR = 1.71, 95% CI: 1.28–2.30; *p* < 0.001)
Dhana et al. (11)	Systematic review and meta-analysis	Cardiovascular mortality	12 eligible studies: 5 studies including 285,675 psoriasis patients, 3 studies including 188,223 patients with mild psoriasis and 4 studies including 17,317 patients with severe psoriasis	Pooled RR = 1.15 (95% CI: 1.09–1.21, *I*^2^ = 65.9%, *P* = 0.02) in patients with psoriasis. Pooled RR = 1.05 (95% CI: 0.92–1.20, *I*^2^ = 90.3%, *P* < 0.001) for mild psoriasis. Pooled RR = 1.38 (95% CI: 1.09–1.74, *I*^2^ = 91.0%, *P* < 0.001) for severe psoriasis
Raaby et al. (10)	Systematic review and meta-analysis	Stroke MI	13 high-quality observational studies	Risk of stroke (HR = 1.10, 95% CI: 1.0–1.19) and risk of MI (HR = 1.20, 95% CI: 1.06–1.35), in patients with mild psoriasis. The risks of both stroke (HR = 1.38, 95% CI: 1.20–1.60), MI (HR = 1.70, 95% CI: 1.18–2.43) and cardiovascular death (HR = 1.37, 95% CI: 1.13–1.67) were increased in patients with severe psoriasis
Pietrzak et al. (22)	Review and meta-analysis	Cardiovascular events	Four case–control and 10 cohort studies.	Elevated risk for CV events in psoriasis patients compared with non-psoriasis controls (OR = 1.28; 95% CI: 1.18–1.38)
Armstrong et al. (97)	Systematic review and meta-analysis	Stroke MI	Nine eligible studies were included representing a total of 201.239 patients with mild and 17.415 patients with severe psoriasis	Risk of MI (RR = 1.29; 95% CI: 1.02–1.63) and stroke (RR = 1.12; 95% CI: 1.08–1.16) in mild psoriasis. Significantly increased risk of cardiovascular mortality (RR = 1.39; 95% CI: 1.11–1.74), MI (RR = 1.70; 95% CI: 1.32–2.18), and stroke (RR = 1.56 95% CI: 1.32–1.84) in severe psoriasis
Samarasekera et al. (98)	Systematic review and meta-analysis	Cardiovascular Disease MI Stroke	Of the 14 included studies, 10 were population-based cohorts, and sample sizes in the psoriasis group ranged from 462 to 130.976	RR relative to the general population was 1.37 (95% CI: 1.17–1.60) for CVD mortality, 3.04 (95% CI: 0.65–14.35) for MI, and 1.59 (95% CI: 1.34–1.89) for stroke
Miller et al. (8)	Meta-analysis	Cardiovascular disease Ischemic heart disease Vascular disease Atherosclerosis Cerebrovascular disease Cardiovascular mortality Diabetes Hypertension Dyslipidemia Obesity Metabolic syndrome	75 studies including up to 503.686 cases and 29.686.694 controls	Cardiovascular disease in total (OR = 1.4; 95% CI: 1.2–1.7), ischemic heart disease (OR = 1.5; 95% CI: 1.2–1.9), peripheral vascular disease (OR = 1.5; 95% CI: 1.2–1.8), and atherosclerosis (OR = 1.1; 95% CI: 1.1–1.2), cerebrovascular disease (OR = 1.1; 95% CI: 0.9–1.3) and cardiovascular mortality (OR = 0.9; 95% CI: 0.4–2.2). Diabetes (OR = 1.9 95% CI: 1.5–2.5), hypertension (OR = 1.8 95% CI: 1.6–2.0), dyslipidemia (OR = 1.5 95% CI: 1.4–1.7), obesity by body mass index (OR = 1.8 95% CI: 1.4–2.2), obesity by abdominal fat (OR = 1.6; 95% CI: 1.2–2.3), and the metabolic syndrome (OR = 1.8; 95% CI: 1.2–2.8)
Miller et al. (99)	Meta-analysis	Total cholesterol LDL Triglyceride Systolic blood pressure Diastolic blood pressure BMI Waist circumference Fasting glucose Non-fasting glucose HbA1c	59 studies with up to 18.666 cases and 50.724 controls	Psoriasis cases had a higher total cholesterol WMD = 8.83 mg dL^–1^, 95% CI: 2.94–14.72, *P* = 0.003. Higher LDL WMD = 9.90 mg dL^–1^, 95% CI: 1.56–18.20, *P* = 0.020. Higher triglyceride WMD = 16.32 mg dL^–1^, 95% CI: 12.02–20.63, *P* < 0.001. Higher systolic blood pressure (WMD = 4.77 mmHg, 95% CI: 1.62–7.92, *P* = 0.003). Higher diastolic blood pressure (WMD = 2.99 mmHg, 95% CI: 0.60–5.38, *P* = 0.014). Higher BMI (WMD = 0.73 kg m^–2^, 95% CI: 0.37–1.09, *P* < 0.001). Higher waist circumference (WMD = 3.61 cm, 95% CI: 2.12–5.10, *P* < 0.001). Higher fasting glucose (WMD = 3.52 mg dL^–1^, 95% CI: 0.64–6.41, *P* = 0.017). Higher non-fasting glucose (11.70 mg dL^–1^, 95% CI: 11.24–12.15, *P* < 0.001) (=0.65 mmol L^–1^ and a Higher HbA1c 1.09 mmol mol^–1^, 95% CI: 0.87–1.31, *P* < 0.001)
Gaeta et al. (100)	Meta-regression analysis	MI Vascular disease Overall mortality Overall Cardiovascular Risk	13 studies. 1.684.032 person-year became available in the psoriasis group and 43.146.770 person-year in the control group	Patients with psoriasis showed an increase of the overall cardiovascular risk compared to the control group (RR = 1.24 [CI: 1.18–1.31]; *P* < 0.00001). Significantly higher risk of infarction (RR = 1.24 [1.11–1.39]; *P* < 0.00001), vascular disease (RR = 1.27 [1.12–1.43]; *P* < 0.00001) and overall mortality (RR = 1.41 [0.97–2.04]; *P* < 0.00001)
Gu et al. (101)	Meta-analysis	Stroke MI Cardiovascular disease Coronary heart disease Peripheral vascular disease Cardiovascular mortality	15 cohort studies	Risk of stroke (RR = 1.26; 95% CI: 1.12–1.41; *p* < 0.0001). Risk of MI (RR = 1.32; 95% CI: 1.13–1.55; *p* = 0.001). Cardiovascular disease (RR = 1.47; 95% CI: 1.30–1.6; *p* = 0.0001). Combined RRs = 1.39 (95% CI: 1.03–1.86; *p* = 0.03) for coronary heart disease, 1.55 (95% CI: 1.02–2.34; *p* = 0.04) for peripheral vascular disease, and 1.33 (95% CI: 1.00–1.77; *p* = 0.05) for cardiovascular mortality
Xu and Zhang (102)	Meta-analysis	Stroke MI	Seven cohort studies	Psoriasis significantly increases the risk of stroke (RR = 1.21; 95% CI: 1.04–1.4) and MI (RR = 1.22; 95% CI: 1.05–1.42) separately. Substantial evidence of heterogeneity was also observed in both subgroup analyses (*P* < 0.001, *I*^2^ = 86.8% and *P* < 0.001, *I*^2^ = 83.1%).

*BMI, body mass index; CAD, coronary artery disease; CCS, coronary calcium score; CI, confidence intervals; LDL, low density lipoprotein; MI, myocardial infarction; OR, odds ratio; RR, rate ratio; WMD, weighted mean difference.*

One crucial point is whether patients with psoriasis have an increased risk of cardiovascular events independently of -versus mediated by- their less favorable risk factor profile. At present, there is evidence for both directions. Prevalence of traditional cardiovascular risk factors is increased among patients with psoriasis compared to non-psoriasis individuals, such as ischemic heart disease, peripheral vascular disease, diabetes, hypertension, dyslipidemia, obesity and metabolic syndrome (8). In addition, several systematic reviews and meta-analyses have established the association of psoriasis with increased rates of atherosclerotic CVD. In a recent meta-analysis by Raaby et al., psoriatic patients, especially those with severe psoriasis, had an increased risk of CVDs (stroke, myocardial infarction, cardiovascular death) (10). Consistent with the above, Dhana et al. showed increased all-cause and cardiovascular mortality risk in psoriasis patients, especially those with severe psoriasis, compared to those without (11). Nevertheless, those with mild disease did not present an increased risk of cardiovascular mortality (11). More recently, increased risk of coronary artery disease was demonstrated in psoriasis by Kaiser et al., assessed by computed tomography and coronary calcium score (CCS) (12). Consequently, available epidemiological data provide solid evidence for a strong link between psoriasis and atherosclerotic CVD, which is at least partially mediated by their aggravated cardiovascular risk profile. Further experimental and clinical studies have attempted to shed light on the primary underlying pathophysiological processes eventually resulting in the establishment of clinically overt CVD independently of traditional cardiovascular factors, as described below.

## Pathophysiology of Endothelial Dysfunction in Patients with Psoriasis: The Role of Chronic Inflammation

**FIGURE 1:**
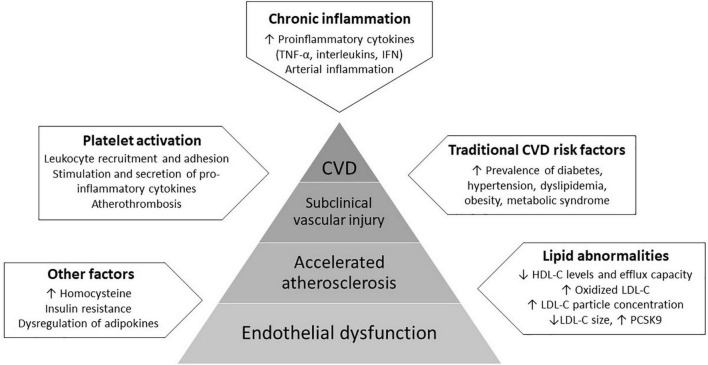
Mechanisms of endothelial dysfunction in psoriasis involve complex interactions between the endothelium, proinflammatory cytokines, circulating lipids and platelets, and the increased prevalence of traditional cardiovascular risk factors. These factors act synergistically directly and indirectly activate endothelial cells and impair their function, resulting in a pro-atherogenic state, the subsequent development of subclinical vascular injury and eventually, clinically evident cardiovascular disease (CVD).

Endothelial dysfunction is defined as loss of vascular dilation in response to biological and mechanical stimuli owing to the pathologic transition of the endothelium into a non-adaptive state secondary to decreased nitric oxide (NO) bioavailability (13). It is considered as a key step in the initiation and progression of atherosclerosis, the development of which involves complex interactions between the endothelium, circulating lipids, platelets, and the immune system (14–19). Several factors, including circulating proinflammatory cytokines, reactive oxygen species, oxidized LDL-C, autoantibodies and traditional cardiovascular risk factors directly and indirectly activate endothelial cells and impair their function, resulting in impaired vascular relaxation, increased leukocyte adhesion, increased endothelial permeability and generation of a pro-thrombotic state (15, 20). Importantly, all of these factors are dysregulated in psoriasis. Due to activation of immune-mediated mechanisms, the vascular endothelium presents a pro-inflammatory phenotype in psoriasis with upregulation of chemotactic, proatherogenic and vascular adhesion molecules, including tumor necrosis factor-α (TNF-α), interleukin-1 (IL-1), IL-6 and the IL-17 family of cytokines, interferon, and vascular cell adhesion molecule 1 (VCAM1). The downstream consequences result in vascular arterial inflammation and direct cytokine-induced injury (20, 21). Mechanisms of endothelial dysfunction in psoriasis are presented in Figure 1.

As the atherosclerotic procedure is being increasingly recognized as an inflammatory process, mechanisms of accelerated atherosclerosis in chronic autoimmune diseases have become a topic of growing interest (22). Pathogenetic mechanisms of CVD in psoriasis appear to be complex and have not yet been fully elucidated. However, psoriasis-triggered pathological pathways, in particular endothelial dysfunction, may activate or augment pre-existing atherosclerosis, which subsequently results in the development of clinically overt CVD (22–24).

Furthermore, chronic inflammation alters lipoproteins structurally and functionally in ways that cannot be captured through standard lipid measurements (25). Inflammation drives modification of LDL-C into small, dense particles that are known to exert pro-atherogenic effects (25). Dyslipidemia is independently associated with endothelial dysfunction, while (26, 27) lipoproteins are implicated in the generation of oxidative stress (26, 28). Oxidized LDL-C modulates NO availability through reduction of eNOS activity or by enhanced metabolism of NO by asymmetric dimethylarginine (ADMA), an endogenous competitive eNOS inhibitor (27, 29, 30). Abnormalities in lipid profile are common in psoriasis and considered to contribute substantially to endothelial dysfunction (31). Patients with psoriasis present reduced levels and efflux capacity of HDL-C, increased LDL-C particle concentration and decreased LDL-C size, as well as elevated levels of circulating PCSK9 when compared to non-psoriasis individuals (31–33).

Platelet activation is regarded as another potent mediator of endothelial dysfunction in psoriasis (34). Apart from their contribution to psoriatic skin lesions, platelets are key regulators of inflammation, immune function, and atherothrombosis. Activated platelets contribute substantially to psoriasis associated inflammation by stimulation and secretion of pro-inflammatory cytokines (18, 35). They perpetuate leukocyte recruitment, adhesion, and rolling along the activated endothelium, thereby promoting atherosclerosis and inducing endothelial dysfunction (36). Patients with psoriasis present increased circulating biomarkers of platelet activation, such as platelet-derived microvesicles, soluble p-selectin, platelet-lymphocyte and platelet-neutrophil aggregates (37). Platelets derived from psoriasis patients augment endothelial cell activation with up to a 20-fold increase in endothelial-derived cytokines such as IL-1β and IL-8 (35). In psoriasis, platelets promote IL-17 secretion from CD4+ lymphocytes, and induce *in vitro* endothelial injury and apoptosis when co-localized with neutrophil subtype granulocytes through the formation of neutrophil extracellular traps, a process known as NETosis (38, 39).

Lastly, although less established, other characteristic features of patients with psoriasis may contribute to the development of endothelial dysfunction. For instance, homocysteine levels are elevated in psoriasis and correlate with the severity of the disease (27). Increased homocysteine promotes oxidative stress and has been associated with the development of atherosclerosis and CVD (40, 41). In addition, insulin resistance is common in psoriasis. As a vasoactive hormone, insulin promotes vasodilation in a NO-dependent manner, increases vasodilation and presents anti-inflammatory effects (42). Likewise, adipokines appear dysregulated in patients with psoriasis, as a result of obesity which is a common comorbidity, but also independently, as they modulate cutaneous inflammation with a probable pathogenetic role in psoriasis (16). Pro-inflammatory adipokines in psoriasis may be released in the peripheral blood as a result of adipose tissue inflammation (16). Elevated adiponectin levels suppress inflammation and immune responses, whereas low adiponectin levels (including leptin, adiponectin, and resistin) are associated with endothelial dysfunction and the development of the metabolic syndrome or CVDs (16).

## Evaluation of Endothelial Dysfunction in Psoriasis

Considering the pathophysiological and clinical significance of endothelial dysfunction, several studies have attempted to quantify the burden of endothelial dysfunction in psoriasis, using both circulating and vascular biomarkers.

### Circulating Biomarkers of Endothelial Dysfunction in Psoriasis

Asymmetrical dimethylarginine (ADMA), oxidized LDL, endothelial progenitor cells (EPCs), endothelial glycocalyx and endothelial microvesicles (EMVs) represent reliable circulating biomarkers of endothelial dysfunction but are currently applied only as research tools (6).

Psoriasis patients present elevated levels of ADMA, a potent eNOS inhibitor of the L-arginine-NO pathway, that correlate with disease severity, suggesting an important role of endothelial dysfunction in the pathogenesis of psoriasis (27). By contrast, ADMA was not increased in a smaller study of mild-to-moderate plaque-type psoriatic patients with low-to-medium grade systemic inflammation (43). Few studies have examined oxidized LDL as a marker of endothelial dysfunction in psoriasis. In a large study of 252 psoriasis patients and controls, psoriasis subjects presented increased levels of lipoprotein (a), oxidized lipoprotein (a) and oxidized HDL (44), although a smaller study of 79 patients with psoriasis and 80 controls failed to reveal any differences in the levels of oxidized LDL (45). Remarkably, oxidized LDL in the former study was significantly associated with non-calcified coronary plaque burden assessed by coronary computed tomographic angiography (44).

Expressed on the endothelial cell surface within blood vessels, the endothelial glycocalyx regulates blood vessel permeability and homeostasis. In a large study of 297 psoriatic patients and 150 controls, glycocalyx thickness in sublingual microvessels was reduced among patients, and correlated with disease activity, carotid atherosclerosis, impaired coronary flow reserve and markers of myocardial deformation assessed by speckle-tracking imaging (46). EPCs are the progenitor cells that are able to differentiate into functional endothelial cells, sustain vasculogenesis and promote vascular repair in ischemic diseases. Significantly reduced levels of circulating EPCs have been measured in psoriasis patients compared to controls, and an inverse correlation with disease severity was observed (47). Likewise, another study recruiting plaque-type psoriasis patients demonstrated decreased EPC levels, as well as an independent association with pulse wave velocity, a well-established marker of arterial stiffness (48). By contrast, these results were not confirmed in a more recent study (17). MicroRNA (miRNA) expression and especially circulating miR-200s were positively correlated with markers of cardiovascular dysfunction such as left ventricular mass (49).

Microvesicles are small vesicles (0.1–1 μm) released from plasma membrane as a result of cellular activation or apoptosis. EMVs display multivalent important biological properties and contribute to vascular homeostasis (50). Their levels increase substantially in patients with CVDs such as hypertension, diabetes mellitus, acute and chronic coronary artery disease (51–53), but also in patients with chronic autoimmune inflammatory diseases (54). Increased levels of EMVs have been found in patients with psoriasis (14, 55). Notably, increased EMVs concentrations in psoriasis were observed beyond cardiometabolic risk factors (55). Another study showed higher ratio of EMVs/EPCs in psoriasis patients, which independently correlated with higher carotid intima-media thickness, an established marker of subclinical atherosclerosis (19, 56). These findings suggest that increased EMVs in psoriasis might not simply represent a consequence of endothelial cell activation, but may also have a role in psoriasis pathophysiology leading to accelerated atherosclerosis.

### Vascular Markers of Endothelial Dysfunction in Psoriasis

The most widely applied, non-invasive vascular methods for the functional assessment of endothelial dysfunction include laser Doppler flowmetry and imaging (LDF/LDI), and the gold-standard flow-mediated dilation (FMD) of the brachial artery, which is currently considered as the gold-standard non-invasive method, with a predictive value for future cardiovascular events especially in high-risk populations (57). Several studies have assessed endothelial dysfunction in psoriasis with FMD, summarized in a recent meta-analysis demonstrating lower FMD measurements among patients compared with controls (56). Although laser Doppler techniques have been mainly applied for the assessment of vascular perfusion within plaques rather than the evaluation of systemic microcirculation in psoriasis (58), NO-dependent vasodilation was attenuated in psoriasis patients in a small study using LDF, and correlated with the degree of psoriatic symptomatology (59). Other vascular methods that focus on the evaluation of endothelial function, such as quantitative coronary angiography and positron emission tomography (PET), are compromised by significant limitations including radiation, reproducibility and cost. However, a recent meta-analysis of 1,427 patients with psoriasis without prior coronary artery disease and 9,670 controls showed higher prevalence and burden of coronary artery disease among patients, detected by CCS with or without cardiac computed tomography angiography (12). Using ^18^F-fluorodeoxyglucose positron emission tomography imaging, a randomized placebo-controlled pilot study showed higher vascular inflammation in ascending aorta of patients with moderate-to-severe psoriasis as compared to controls (60). Similarly, arterial inflammation was more pronounced in patients with mild psoriasis compared to controls by use of the same method (61).

Collectively, available data regarding the above circulating and vascular biomarkers appear in line with the hypothesis that endothelial dysfunction is implicated in the development of accelerated atherosclerosis in psoriasis. Further extending this notion, the potential improvement of endothelial function following successful control of psoriasis-related inflammation has been the subject of several studies over the past years.

## The Impact of Pharmacological Interventions on Endothelial Dysfunction in Psoriasis

To date, topical therapies are the cornerstone for managing mild psoriasis which typically covers less than 5% body surface area (62). However, patients with moderate and severe disease, who are presumably at higher cardiovascular risk based on the above, are candidates for newer systemic therapies as first-line treatment (63, 64). The observation that these therapies could target the accompanying vascular dysfunction and ameliorate biomarkers of cardiovascular risk (65), has several therapeutic implications for cardiovascular risk prevention in psoriasis that are currently under vigorous investigation.

### Tumor Necrosis Factor-α Inhibitors

Tumor necrosis factor-α inhibitors (adalimumab, certolizumab, etanercept, or infliximab) showed a protective cardiovascular profile in multiple, mainly observational studies of psoriasis. It has been hypothesized that this action is probably mediated by their beneficial effect on endothelial cell function (66). There is at present no strong, definite evidence for a significant beneficial effect of anti-TNF-α biologics on endothelial function in psoriasis (66, 67), although some promising data do exist. TNF-α blockade has led to improvement of vascular function assessed through resting endothelium-dependent vascular tone by low-flow-mediated constriction, yet FMD values remained unchanged (68). In this context, treatment with TNF-a inhibitors was associated with significant reductions in endothelial and platelet microvesicles levels (69). A small cohort study detected a significant improvement of endothelial dysfunction markers (serum intercellular adhesion molecule 1 and FMD) after a short-period treatment with adalimumab (70). Adalimumab has shown anti-inflammatory effects and improved FMD in patients with psoriasis (71). Conversely, a recent RCT study, that used 18F-FDG PET-CT to assess vascular inflammation, did not detect any superiority of adalimumab over phototherapy or placebo (72). In a recent meta-analysis of RCTs, there was no beneficial effect on imaging biomarkers (aortic vascular inflammation or FMD) of cardiovascular risk in patients exposed to adalimumab (73). Treatment with etanercept increased the EPC count in a small double blind, placebo-controlled, cross-over study, indicating improved endogenous endothelial regenerative capacity, but brachial artery flow-mediated and nitroglycerin-mediated dilation was not modified with treatment (74).

### IL-17 and IL-23 Inhibitors

IL-17 inhibitors target either the IL-17 ligand (secukinumab, ixekizumab, and bimekizumab) or its receptor (brodalumab). Secukinumab did not show any clinically significant effect on aortic vascular inflammation (assessed by 18F-FDG PET/CT) in a placebo-controlled RCT either on short- or long-term follow-up (75). Nevertheless, the CARIMA (Evaluation of Cardiovascular Risk Markers in Psoriasis Patients Treated with Secukinumab) trial indicated the protective role of secukinumab on endothelial cell function measured with FMD (76). Recently, the potential benefit of a reduced dose interval has been examined in heavier patients and in those with suboptimal responses (77, 78). Ustekinumab inhibits both IL-12 and IL-23 by targeting their shared p40 subunit. Positive effects of ustekinumab impact on vascular inflammation were observed using 18F-FDG PET/CT (79). However, levels of circulating endothelial- and platelet-derived microvesicles remained unchanged in patients with psoriasis successfully treated with anti-IL-12/23, regardless of clinical improvement (80). In a recent meta-analysis of RCTs, ustekinumab, yet not secukinumab, induced short-term reductions in aortic vascular inflammation, whereas FMD remained unchanged. Nevertheless, these reductions were not sustained in the long-term (73). The hypothesis of cardiovascular risk reduction in psoriasis patients receiving ustekinumab (81) was further questioned by the observation of no substantially different risk of major adverse cardiovascular events among TNF-a inhibitors, ustekinumab or placebo therapy in several studies (82–85). The effect of newer IL-23 and other cytokines inhibitors recently approved (Guselkumab, Tildrakizumab, and Risankizumab) on endothelial dysfunction remains to be investigated (86–92).

### Oral Systemic Treatments

In a prospective longitudinal pilot study, systemic therapy with fumaric acid esters resulted in an improvement of endothelial vasodilator function assessed by venous occlusion plethysmography (93). Methotrexate, apremilast, acitretin, and cyclosporine are oral available treatment options for psoriasis. Nevertheless, evidence regarding protective effects on cardiovascular disease in psoriasis exists mainly for methotrexate (94, 95), which has shown neutral short-term effects on endothelial function in patients with psoriasis (96). Further studies are warranted to determine the effects of these treatment modalities on endothelial dysfunction in patients with psoriasis.

## Conclusion and Future Perspectives

Psoriasis, especially in its severe forms, is an independent risk factor for cardiovascular morbidity and mortality. Several psoriasis-induced mechanisms promote the development of endothelial dysfunction, which acts synergistically with chronic inflammation and activates or potentiates pre-existing atherosclerosis in psoriasis. Available human studies using the most widely applied circulating and vascular biomarkers of endothelial dysfunction provide clinical evidence of endothelial dysfunction in patients with psoriasis, that correlates with disease severity. However, the clinical utility of biomarkers of endothelial dysfunction in psoriasis patients in terms of cardiovascular risk prediction needs to be addressed further. Moreover, it is hypothesized that effective control of the disease might improve endothelial cell function and subsequently modulate cardiovascular risk. Although some encouraging data have been published, large, prospective, appropriately designed studies are urgently warranted to provide strong evidence regarding the possibility of sustained beneficial effects on endothelial function of such therapies in psoriasis. Last but not least, future studies need to investigate whether interventions specifically targeting at the improvement of endothelial function in patients with psoriasis might provide incremental benefits in the modulation of both chronic inflammation and risk of future CVD.

## Author Contributions

EG, EL, and AP contributed to the conception and design of the study. PA and AM performed the literature searching and wrote the first draft of the manuscript. KG and MG wrote the sections of the manuscript. KG prepared the table. PA designed the figure and edited the final draft of the manuscript. EG reviewed and supervised the final version of the manuscript. All authors contributed to manuscript revision, read, and approved the submitted version.

## Conflict of Interest

The authors declare that the research was conducted in the absence of any commercial or financial relationships that could be construed as a potential conflict of interest.

## Publisher’s Note

All claims expressed in this article are solely those of the authors and do not necessarily represent those of their affiliated organizations, or those of the publisher, the editors and the reviewers. Any product that may be evaluated in this article, or claim that may be made by its manufacturer, is not guaranteed or endorsed by the publisher.
